# The Sun-Earth connect 3: lessons from the periodicities of deep time influencing sea-level change and marine extinctions in the geological record

**DOI:** 10.1186/s40064-015-0942-6

**Published:** 2015-06-20

**Authors:** Robert GV Baker, Peter G Flood

**Affiliations:** Geography and Planning, University of New England, Armidale, 2350 Australia; Earth Sciences, University of New England, Armidale, 2350 Australia

**Keywords:** Periodicity, Geological record, Solar model, Self-similarity

## Abstract

A number of papers since Rampino and Stothers published in *Science* 1984 have reported common periodicities in a wide range of climate, geomagnetic, tectonic and biological proxies, including marine extinctions. Single taper and multitaper spectral analysis of marine fluctuations between the Late Cretaceous and the Miocene replicates a number of the published harmonics. Whereas these common periodicities have been argued to have a galactic origin, this paper presents an alternative fractal model based on large scale fluctuations of the magnetic field of the Sun. The fluctuations follow a self-similar matrix of periodicities and the solutions of the differential equation allow for models to be constructed predicting extreme events for solar emissions. A comparison to major Phanerozoic extinction, climate and geomagnetic events, captured in the geological record, show a striking loop symmetry summarised in major 66 Ma irradiance and electromagnetic pulses from the Sun.

## Introduction

The proposition that human emissions of greenhouse gases (GHGs), especially carbon dioxide, has and will continue to produce warming of the globe and accelerating sea-levels is the current paradigm. The claim is that anthropogenic input of GHGs is so large that we can assign the term “Anthropocene” Epoch for the present because it is dominated by human-induced effects. The predictions are accepted unequivocally with the end result being that climate change is the defining issue of today.

However, Anthropogenic Global Warming (AGW) should also be considered in the context of the geological record of climate change and its proxies, such as sea-level fluctuations, for it is now accepted that during the past two billion years the Earth’s climate has fluctuated between “Icehouse” (Cold) periods and “Greenhouse” (Hot) periods. For example, Miller et al. (2008) describe the largest global cooling event of the Cenozoic (33.8 and 33.5 Ma ago), where the Earth’s climate switched from warm, high CO_2_ conditions, to variable “Icehouse” climates where icesheets grew to be 25% larger than present. There was a corresponding ~67 m eustatic sea-level decrease and there was a 21% extinction of taxa in the geological record (Sepkoski 2002). Such fluctuations have been argued not to be random but periodic. Raup and Sepkoski (1984) analysed ~3,500 families of marine animals (vertebrate, invertebrate and protozoan) and concluded that there was significant periodicity in 12 extinction events with a 26 Ma mean interval. More recently, Rohde and Muller (2005) using Fourier Spectral Analysis (FSA), identified a strong periodicity of 62 ± 3 Ma in the cyclicity of marine diversity through the Phanerozoic, based on an analysis of Sepkoski (2002) compendium. Lieberman and Melott (2012), using the Paleobiology Data Base (PBDB), undertook spectral analysis of this data base, which also showed a strong spectral peak of 63 Ma. With such periodicities a recurring result in data analysis, further discussions have eventuated on the origin of this periodicity, focusing on the position or motion of the Sun moving through the Milky Way (Lieberman and Melott, 2012).

Smith and McGowan (2005) have contested the biological implications of this periodicity, arguing that the fossil record mirrors the rock outcrop area and that the cyclicity comes from sampling rather than biological signals. However, Omerbashich (2006) used Gauss-Vanicek spectral analysis of the Rohde and Muller (2005) data, removed all zero values and still found significant periodicities at 140.23 Ma and 91.30 Ma at 99%; and 110.3 Ma, 66.85 Ma and 32.12 Ma at 95% confidence levels. Melott (2008) undertook power spectrum analysis using Lomb-Scargle methods and found significant periodicities of 99.9% at 63.1 ± 6 Ma and 46 Ma and argued that the 62 Ma periodicity appears in two largely independently generated data sets with multiple methods of analysis (Table 1). This periodicity has been further re-analysed and examined by Lieberman and Melott (2012) using the PBDB, which again showed a strong peak of 63 Ma with the spectral peaks differing by only 1.6 Ma. They conclude that there is a strong signal within paleobiological data bases of 62 ± 3 Ma and 31 ± 1 Ma and a specific extinction metric of 27 ± 1 Ma, despite criticisms of individual data bases, analytical methods and problems of sampling from the fossil record.

**Table 1:** Periods (in millions of years) corresponding to the highest peaks in the spectral analysis of six Phanerozoic time series (after Rampino and Stothers 1984; Rohde and Muller 2005; Omerbashich 2006) and compared to Row 3 and Row 5 harmonic predictions from the Sun (L = loop; LT = loop and tail;and L2T = loop and double tail)

**Marine diversity**	**Impact craters (N = 65)**	**Tectonic episodes (N = 18)**	**Carbonatite intrusions (N = 28)**	**Kimberlite intrusions (N = 38)**	**Geomagnetic reversals (N = 24)**	**Solar harmonics R3 (fast)**	**Solar harmonics R5 (slow)**
	**12**	**12**	**13**	**12**	**12**	**11.49 L**	**13.18 L**
	**16**	**16**	**16**	**16**	**15**	**14.37 LT**	**16.48 LT**
	**20**	**20**	**23 ± 4**	**23**	**21**	**17.24 L2T**	**19.77 L2T**
**32.1**	**32 ± 1**	**33 ± 3**	**34 ± 5**	**35 ± 1**	**33 ± 1**	**34.47 L2T**	**32.96 LT**
**46**	**49**	**44**			**47**	**45.97 L**	
			**50 ± 3**	**56**			**52.73 L**
**63 ± 3**	**61**	**61**		**68**	**63**	**68.96 L2T**	**65.92 LT**
**91.3**	**96**		**90**			**91.94 L**	
**110.3**					**114**	**114.93 L2T**	**105.47 L**
**140.2**						**137.19 L2T**	**131.83 L**
	**260**	**270**	**235**	**280**	**285**	**275.74 L2T**	**263.67 LT**

Rampino and Stothers (1984) summarise the spectral peaks of five Phanerozoic time series (impact craters, tectonic episodes, carbonatite intrusions, kimberlite intrusions and geomagnetic reversals; Table 1). There were common periodicities of 12 Ma, 16 Ma, 20-23 Ma and 32-35 Ma. Further, Rampino & Stothers (1984) analysed the periodicity in Vail et al. (1977) Exxon sea-level data and found two periodicities in the spectrum of residuals of ~21 Ma and ~33 Ma. Likewise, analysis of major discontinuities of sea-floor spreading produced periodicities of 18 Ma, 23 Ma and 34 Ma. Active tectonism on the continents also correlates with episodes of lower sea-levels. They also analysed pulse phases of 18 principal Phanerozoic orogenic phases revealing spectral peak clusters of 20 Ma and 31 Ma to 33 Ma, 36 Ma, 44 Ma, 61 Ma, 81 Ma and 270 Ma. Geomagnetic reversals follow similar periodicities (Table 1). What could have produced the concurrence in these cycles? Rampino & Stothers (1984) conclude various external forcing mechanisms such as collisions with comets and asteroids as the likely cause with crater periodicities of 12 Ma, 16 Ma, 32 Ma and 260 Ma, but there still needs to be an underlying periodic mechanism to account for such correlations, such as, between orogenic events and biological extinctions. The key question remains in the understanding of the dynamics within the geological record: why do such a plethora of proxies produce such common periodicities?

## Sea-level change in the Phanerozoic

### Background

Sea levels are the manifestation of the sum totals of climate change parameters including forcing and feedback factors. At present values of 460 ppm C0_2_-equivalent, the Earth’s climate is tracking towards the upper stability limit of the Antarctic ice sheet which is defined at 500 ppm and 4°C warmer than present. The resulting melting would raise sea levels by 4-6 m (as was the case in the last Pleistocene interglacial 126,000 yr BP). Above this level of 500 ppm, the Earth would track from its current Icehouse conditions back to Greenhouse Earth conditions, such as, during the mid-Eocene 40 Ma ago with the consequences of substantial sea-level shifts and fundamental climate change.

**Figure 1:**
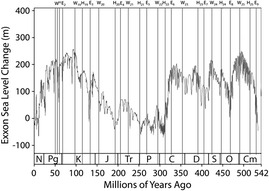
The Exxon Sea-level Curve with E, H and W solar phase boundaries (see Figure 4). W-peaks are warm period maximums from the intense irradiance of an active Sun, where E-points and H-points are the beginning and end boundaries for the passive phases of the Sun. H-phases relate to glacial periods and lowstands in Phanerozoic sea levels, whereas W-phases relate to high sea surface temperatures and highstands in sea level.

Research during the past four decades has established certain relationships between sea level, ice volumes, temperature and carbon dioxide (Miller et al. 2005; Haq and Schutter 2008). The global or eustatic sea-level changes are principally controlled by two variables, namely, the volume of water in the oceans, and the volume of ocean basins. The Earth’s sea-level record for the past 540 Ma has been summarised by Hallam (1984) and Haq et al. (1987). The publication of the record by Vail et al. (1977) and Haq et al. (1987), including sea-level histories, which in industry circles is referred to as the Exxon Production Research (EPR) record, represents a major achievement in Earth Sciences (Figure 1). Most of periodicities observed in spectral analysis of sea levels and sea-floor spreading again show similar periodicities to Table 1 of between 18-23 Ma and 33-34 Ma (Rampino & Stothers, 1984) suggesting complex climatic-tectonic interdependence.

### Spectral analysis of sea-level fluctuations

Sea-level fluctuations can now be analysed by sophisticated spectral analysis using a recent detailed data from the Late Cretaceous to Miocene (108–9.7 Ma) (Kominz et al. 2008). Two methods of analysis will be used; firstly, a single taper Blackman (Harris) spectral analysis to discern longer cycles in the record and secondly, multitaper spectral analysis (Thomson, 1982) using five tapers (K = 5) and a bandwidth (BW = 4) to deconstruct any shorter cycles within the data set. Multitaper methods render power spectrum analysis using Lomb-Scargle single taper methods, such as in Melott (2008) as only, at best, first order approximations.

Spectral methods, such as, the Lomb-Scargle algorithms, over-emphasise data points at the centre and weakly weights extreme values. The Blackman tapers can apply 20% cosine weightings (or tapers) to address this problem and discards only 12.5% of available data variable constraints. This taper may be adequate in many cases but would not be appropriate for dispersive or unusually band-limited signals (Park et al. 1987). It can, however, analyse unevenly partitioned data: a common feature of climate and geological proxies. The Multitaper spectral method was developed by Thomson (1982) to overcome the trade-off between the resistance to spectral leakage and the variance of the spectral estimates from single taper algorithms. It discards very little data and weights the data relatively evenly with significance determined by an F-Ratio and therefore is quite sophisticated (Park et al. 1987). The data partitioning over time, however, must be evenly spaced.

The Blackman (Harris) algorithm was undertaken on the Kominz et al. (2008) evenly partitioned data set using *Autosignal* ‘Blackman-Harris 4’ (BH4) with a cosine taper, whilst the multitaper analysis (BW = 4 and K = 5) used *Autosignal* for ‘Fourier Multitaper Spectra’. The data samples are every 100 ka meaning that both methods were appropriate. The significant periodicities are listed in Table 2. The Blackman (Harris) BW4 algorithm produced significant periodicities at 143.55 Ma and 31.16 Ma at 99% and 17.07 Ma at 95%. The Multitaper analysis, where significant F-values (95%) must be coincident with spectral peaks (dB), yielded shorter periods of 724,482; 1,040,736; 2,059,639; and 4,886,962 yrs (Table 2). The increased number tapers deconstruct the longer periods from BW4 and are less useful to compare with the summary results in Table 1. The sea-level periodicities therefore share common signals of 16 ± 1 Ma and 34 ± 2 Ma and 140 Ma (half the 275 ± 10 Ma) with periodicities in marine diversity, tectonic episodes, intrusions and geomagnetic reversals. Sea-level change is therefore also part of this synchronicity.

**Table 2:** Spectral Analysis of the Kominz et al***.*** 2008 data on sea-level fluctuations from 9.7 to 108Ma (at ** 99%; * 95% and 90% significance)

**Blackman (Harris) BH4**			**Multitaper BW = 4 K= 5**				**Theoretical loop (L), loop & tail (LT) and loop & double tail (L2T) periods**	
**Frequency**	**Power**	**Period (Ma)**	**Frequency**	**dB**	**F-value**	**Period (yr)**	***Solar Harmonics R3 (fast)***	***Solar Harmonics R5 (slow)***
**0.00698**	133.35**	**143.55**	**1.38030**	**50.11**	8.996**	**724,482**	**718,336**	**NA**
							**L**	
**0.03209**	18.80**	**31.16**	**1.43889**	**47.90**	8.790*	**694,978**		
**0.05860**	10.51*	**17.07**	**0.96081**	**52.19**	8.154*	**1,040,786**	**1,077,504**	**1,029,971**
							**L2T**	**LT**
			**1.82489**	**45.92**	7.002*	**547,977**		
			**0.48552**	**55.04**	6.393*	**2,059,639**	**2,155,008**	**2,060,000**
							**L2T**	**LT**
			**0.63620**	**46.51**	5.099*	**1,571,830**		
			**3.25914**	**46.22**	4.832*	**306,829**		
			**0.20462**	**58.86**	4.730*	**4,886,962**	**4,310,016**	**4,944,000**
							**L2T**	**LT**
			**3.45539**	**47.74**	**4.372**	**289,403**		
			**1.22590**	**51.23**	**4.306**	**815,729**	**NA**	**823,858 L**
				**46.51**	**4.207**	**1,478,860**	**1.436,672 L**	**1,648,000 L**
				**45.71**	**4.193**	**536,760**	**538,752**	**514,997**
							**L2T**	**LT**

### Spectral analysis of marine stratigraphic variability

The tectonic episodes follow the same periodicity as the other proxies. Recent research by Myers and Peters (2011) of stratigraphic variability in North America during the Phanerozoic using Multitaper Spectral Analysis confirms this with a strong periodicity of 56 ± 3 Ma. This is concurrent with the ~50 Ma periodicity in magmatic activity in the Sierra Nevada batholith. They argue that this timing is consistent with other oscillatory proxies and its tempo is statistically similar to known rhythms in a number of marine animal genera in the global fossil record. Meyers and Peters further demonstrate that there is a eustatic contribution where marine strata in North America exhibits 55 Ma oscillations.

### What is the origin of this periodicity?

Extra-terrestrial sources have been suggested to explain the periodicity, where Raup & Sepkoski (1984) propose that the path of the Sun through the spiral arms of the Milky Way, affects the cosmic ray intensity reaching the Earth. Shaviv (2003) analysed 50 iron meteorites and deduced that there was a 143 Ma periodicity in the cosmic ray intensity, which is one of the frequencies of marine diversity and sea-level fluctuation in the spectral analyses. More recent hypotheses are summarised by Lieberman and Melott (2012) who look at possible origins from the motion of the Sun within The Milky Way with a 200 Ma approximate period from a wobble of the Sun in transit, coinciding with a vertical oscillation of 63 Ma. Whilst such explanations are possible, they are still not convincing, since they do not explain the appearance of equally justifiable and possibly related sub-harmonics in the spectral analysis. The key question remains: why do such a plethora of proxies produce such common periodicities?

**Figure 2:**
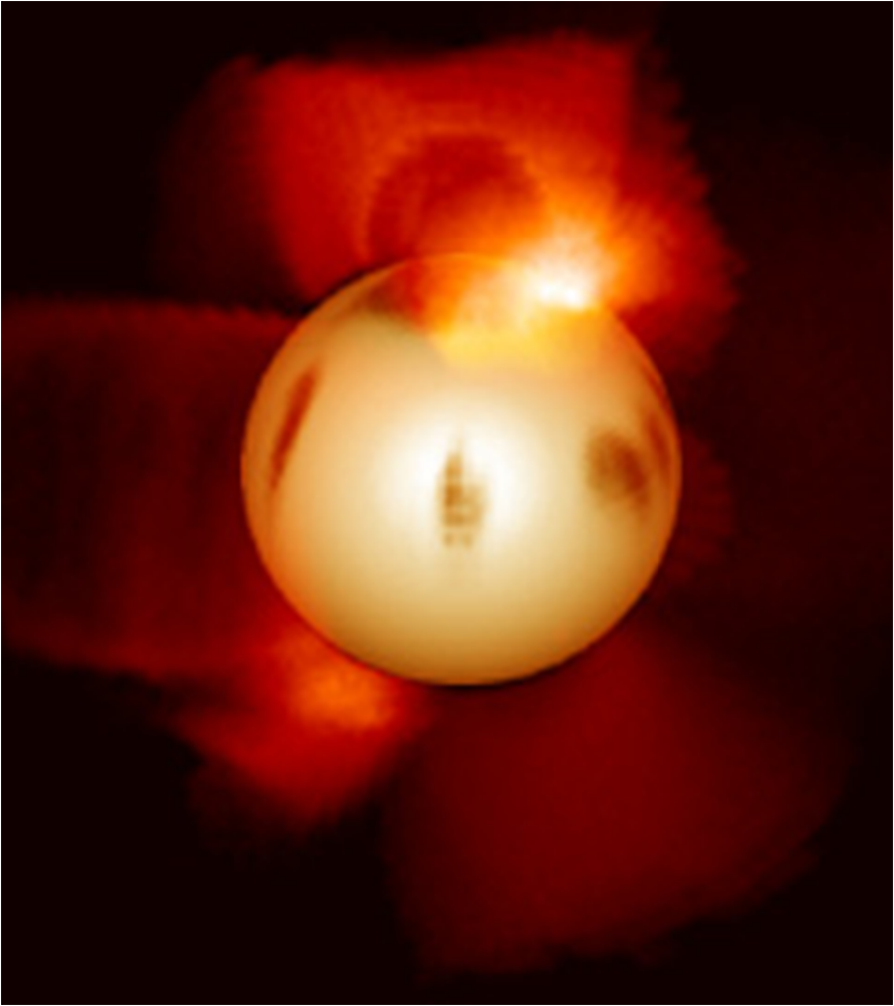
A computer simulation of the star AB Doradus where the starspots form along meridians of longitude and the polar zones are regions of sunspot and solar flare maximums. Does the Sun flip to such magnetic field structures during higher rotational phases and extremes of thermonuclear production, synchronously affecting the Earth’s geomagnetic field and atmospheric ionisation? (Source: Cameron A., Jardine M., Wood K.,University of St Andrews)

Another major external source could be concurrent large-scale periodic fluctuations in the Sun’s magnetic field and irradiance affecting the Earth’s geomagnetic field and climate systems. Such extreme fluctuations could involve in-phase flipping between the latitudinal and longitudinal twisting in the Sun’s toroidal and meridinal magnetic fields. The flipping would depend on the Sun’s core rotational speed and whilst sunspot formation occurs latitudinally at present, other stars, such as, AB Doradus or EK Dranconis, have starspots forming along meridians of longitude and the poles are a convergence of sunspot formation (Figure 2). This flipping mechanism could ensure long term relative stability in irradiance output within a chaotic system of thermonuclear production (Baker 2014). Such flipping behaviour in the Sun’s dynamo in the core and the changing location of field-line emissions and solar flare production could synchronously affect ionization of the Earth’s atmosphere, impacting on global temperatures, particularly at the poles. Further, the Sun’s magnetic field switching through a cycle of situations could underpin the Earth’s geomagnetic field reversals, producing fundamental shifts in convective currents and plume locations in the mantle, and in the transitional phases, increasing the incidence and severity of UV radiation and galactic cosmic ray penetration to land and ocean surfaces. Such a source could explain the coincidence in periodicities, if they could be shown to follow the same long-term periodicities within a pulsating Sun.

If the Sun’s magnetic field flipping coincides with the geomagnetic reversals on Earth, there is little geomagnetic field protection during the switching process of between 5000–10,000 yr and the Earth will concurrently receive high UV-B emissions and galactic cosmic ray incursions during these transitions between field flipping and rotation of the solar dynamo within thermonuclear production. The effect of UV-B radiation of phytoplankton production rates is significant (Larsen, 2005), so such saturation would have the ability to significantly disrupt marine food webs as well as terrestrial species. This would also be a time of high galactic cosmic ray incursions and atmospheric ionization. If there were concurrent changes to the Sun’s gravitational pull on the solar system, it would not be surprising that impact crater periodicities could be largely synchronous with geomagnetic reversals, sea-level fluctuations and marine extinctions (Table 1).

## The Sun as a pulsating star

### A self-similar matrix of solar harmonics

“An additional geological source of solar history that remains largely untapped hinges on the shifts in the global atmospheric circulation that appear to result from cyclical fluctuations in irradiance” Vita-Finzi (2011).

There appears to be a number of cycle periods linked to solar output. Not only are there ~27-day and ~11-year cycles, but research from isotopic analysis of the Greenland Ice Core also suggests a ~1400 year cycle (Bond et al. 1997). There is a common ratio between these three cycles which turns out to approximate the inverse of the fine structure constant α (or 1/α = 137.0356), the fundamental constant of electromagnetic radiation. A matrix of possible harmonics can be constructed based on the fine structure constant maintaining the solar ‘constant’ (Baker, 2014, Table 3). The initial column (column *i*) is a sequence of 27.275d, 10.24 yr, 1403 yr, …, and arbitrary harmonic rows to the right (*n* = 2, 4, 8…) and to the left (*n* = 1/2, 1/4, 1/8 …) (Table 3). The initial column contains a sequence within the range of the familiar ~10.24 yr sunspot cycle and an ~1403 yr Bond Cycle; and in the column *j*/row 2 (R2) elements, a magnetic Hale Cycle at 20.48 yrs and the column *k*/row 3 (R3) elements, a Gleissberg Cycle at 81.92 yr. The diagonals have an inversion between the multiplicative feature of *2n*/*α* periods to the right for an active Sun and *n*/*2α* to the left for a passive Sun (or *4n*, *n* respectively). The range for each of these cycles is defined by the values obtained from each row relative to fast or slow rotations of the Sun (that is, for Sunspot Cycle, Row 2 = 10.24 yr; Row 3 = 10.94 yr; Row 4 = 11.71 yr; and Row 5 = 12.56 yr, ………). The theoretical range of the Sunspot Cycle is 10.24 to 12.56 yr, yet this is what has been measured in the historical record, where the average sunspot cycle is 11.1 yr, yet the mode is 10.25 to 10.75 yr and there is also a further peak between 11.25 and 12.75 yr (http://www.ips.gov.au/Educational/2/3/7: accessed 31/7/2013). The matrix predicts that this is not a random fluctuation, but part of a cycle of situations that the Sun enacts oscillating between these elements of the matrix rows. The R3 row appears in this context to be the ‘average’ harmonic for the sequence of situations for this pulsating Sun. The fractal assumption is that self-similarity is replicated across all time scales around a fixed energy axis.

**Table 3:** **Theoretical loop periods (L) predicted by a matrix of possible rotational core and tachocline harmonics partitioned by the inverse of the electromagnetic fine structure constant (α =1/137.0356)**

	**Col -** ***p***	**Col -** ***m***	**Col ** ***-l***	**Col ** ***-k***	**Col -** ***j***	**Col ** ***i***	**Col ** ***j***	**Col ** ***k***	**Col ** ***l***	**Col ** ***p***
Row 1		1.71d	3.41d	6.82d	13.64d	27.275d	54.55d	109.1d	218.2d…	[9.56 y]
						*Carrington*				
						*Rotation*				
						***r, 0***				
Row 2	Col -***p***	0.64y	1.28yr	2.56y	5.12y	10.24y	20.48y	40.97y	81.92y	1310y
	(29.2d)					*Fast Sunspot*	Hale		*Fast Gleissberg*	*Fast Bond*
						***n,*** (1/α)				
Row 3	Col-p	88y	176yr	351y	702y	1403y	2806y	5612y	11,226y	718,336y
Col -***w***	10.94y	*Gleissberg*				*Bond*				Average
(31.2d)	*Sunspot*					***m,***(1/α^2^)				Pleistocene
Row 4	1499y	12,021y	24,042y	48,106y	96,213	192,426	384,852	769,704y		
(11.71y)						***q,***(1/α^3^)		Pleistocene		
*Slow Sunspot*	*Slow Bond*									
Row 5	823,858y	1.648Ma	3.296Ma	6.59Ma	13.18Ma	26.367Ma	52.73Ma	105.45Ma	210.94Ma	
(12.56y)	*Slow*					***z,***(1/α^4^)				
*Sunspot*	*Pleistocene*									

### The physical model

Solar self-similarity described in the matrix (Table 3) has been modeled physically in Baker (2014) and the solutions of the differential equation are required to determine the nature of solar emissions in this dynamic model. Key outcomes are summarized below to help understand the nature of extreme fluctuations at phase boundaries in solar emissions.

- The postulate is that any real scale functions *L(x)* of a dependent solar variable S and another independent phase variable *Ω (x)* of a variable *t*, is such that for any two positions *x*_*1*_ and *x*_*2*_ in solar locations, there exists two times *t*_*1*_ and *t*_*2*_ where the solutions ‘look alike’ after a rotation. The repeated pattern of the time-space sunspot and polarity butterfly diagram is an example of this time-scaled similarity assumption. The ratios must have the same numerical value for all pairs of values at *t*_*1*_ and *t*_*2*_, namely: *S*(*t*_1_, *x*_1_)/*L*(*x*_1_) = *S*(*t*_2_, *x*_2_)/*L*(*x*_2_) = *nΩ* = (1/*α*^*R* − 1^) for *n* = …, *1*/*4, 1*/*2, 1, 2, 4, 8,*…; where *R = 1, 2, 3*, *4*.. . rows and *α* is the fine structure constant. The solar variable has the form S*(t ,x)* = *L*(*x*) K(*η*) where *η = t* /*Ω (x*) and is a time-scaled phase variable across a partitioning of time. The time phase similarity is defined by a fractal (1/*α*^*R* − 1^) and the matrix in Table 3 for *T* fractal periods is *nΩ*_*0*_(*x*) × (1/*α*^*R* − 1^) = *T* for the ‘average’ Carrington Rotation *Ω*_*0*_(*x*) =27.275d. The corollary of this assumption is that the energy of the Sun is the same for all pairs of values between surface locations (that is, there is a ‘fixed energy’ in irradiance) and that time ‘loops’ around this fixed energy axis.
- The self-similarity differential equation is of the form

1 $$ \frac{d^2K}{d{\eta}^2}+2\kappa n\eta \frac{dK}{d\eta }-\beta K=0 $$

with two conditions, firstly

2 $$ {\varOmega}^2(t)={\varOmega}_0+4\kappa Dx $$

and secondly

3 $$ L(x)=\gamma {\left({\varOmega}_0^2+4\kappa Dx\right)}^{\beta /4\kappa } $$

- The interpretation of the constants γ, β, *Ω*_*0*_ , and κ, define a four stage zonal model of the Sun, where they can either be zero, real and non-negative or imaginary, namely:

- *Situation 1: ( κ = 0, β = −λ*^*2*^*) Solar Core,*
- *Situation 2: ( κ > 0, r bounded) Radiative Zone,*
- *Situation 3: ( κ < 0, R bounded) Tachocline,*
- *Situation 4: ( κ > 0, R unbounded) Corona.*

- For the initial energy distribution we are interested in the solutions from the radiative zone (κ > 0, r bounded) from the differential equation:

4 $$ \frac{d^2K}{d{\eta}_b^2}+2{\eta}_b\frac{dK}{d{\eta}_b}-\frac{\beta }{\kappa }K=0 $$

The only bounded solution from emissions from the radiative zone is when the constant *β*/*κ* is an even integer 2*n*. For κ > 0 in real time and during a stable Sun, the solution of irradiance distribution *S* from the radiative zone is in the form of iterated error functions as:

5 $$ S\propto {\left(4D{R}_{\ast}\right)}^{n/2}{i}^n erfc\left(\pm t/2\sqrt{D{R}_{\ast }}\right)\forall n\ge -1 $$

**Figure 3:**
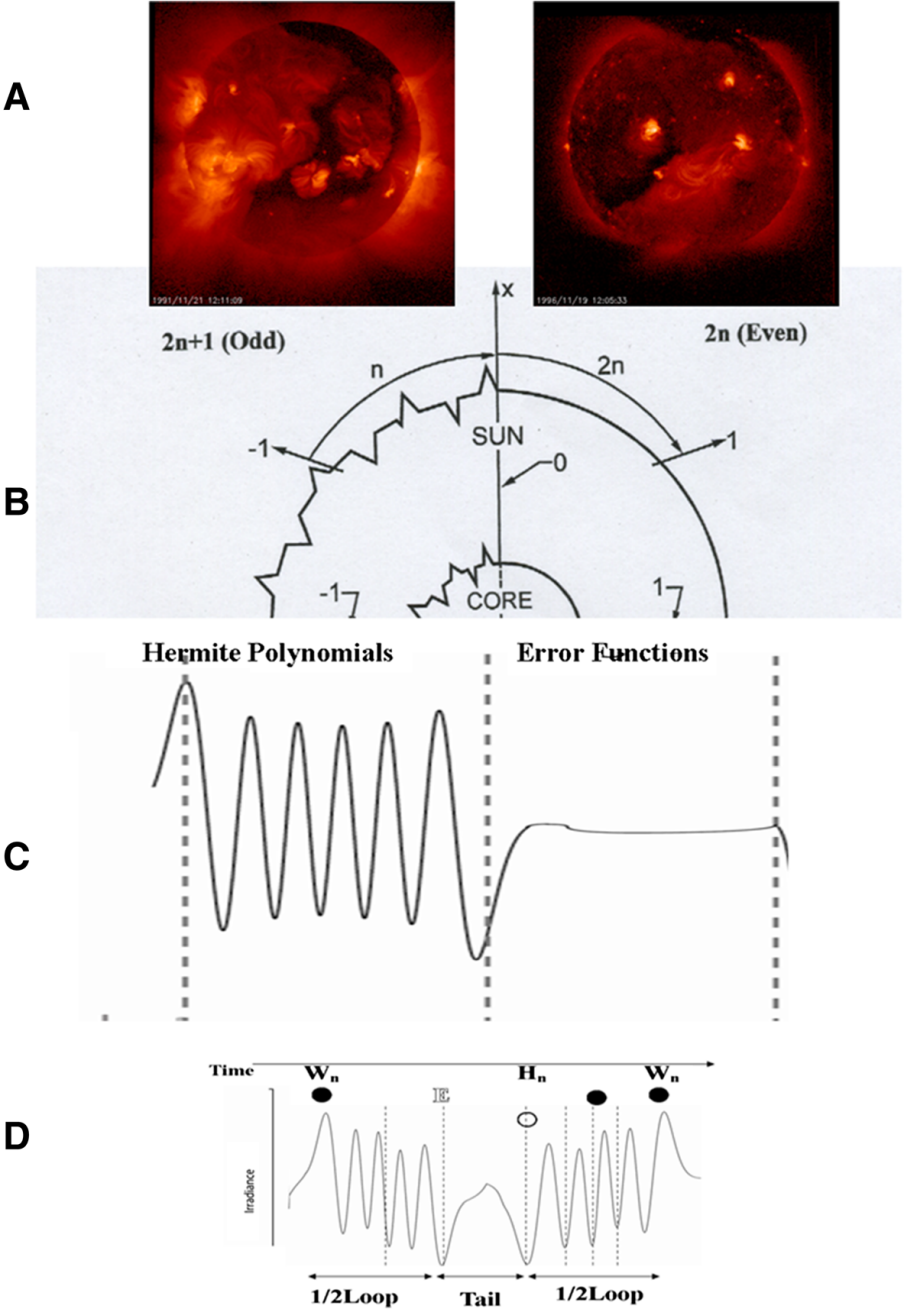
**A** The Sun flips from an active state with sunspots and solar flares to a passive Sun. **B** The solutions to the self-similar DE are defined for *2n + 1* for an active Sun and *2n* for a passive Sun. **C.** They take the form of either a decaying Gaussian multiplied by Hermite polynomials *n* = 1, 3, 5,… for an active Sun, analogous to a quantum harmonic oscillator or iterated error functions for a passive Sun. **D** The extremes in irradiance in the loops of the Hermite polynomials are **W**-events where the magnetic field flips. The beginning and end of the polynomials which are mirror images define the tail. The first global minimum is defined as an **E**-event and the second global minimum beginning the next cycle an **H**-event where there is a solar minimum and usually temperatures are lowest in the sequence.

This defines the irradiance distribution from a passive Sun during the period of the sunspot minimum (Figure 3A, B).

- The twisting at the tachocline from rotational shear means that *L* can be imaginary in magnetic tube rotations of the first subsidiary condition and the new equation is the well-known Hermite differential equation used to describe a harmonic oscillator in quantum mechanics, namely:

6 $$ \frac{d^2K}{d{\eta}_b^2}-2{\eta}_b\frac{dK}{d{\eta}_b}+\frac{\beta }{\kappa }K=0 $$

The solution of the differential equation, therefore, is of the form of a decaying Gaussian multiplied by Hermite polynomials *n* =1, 3, 5,…, namely:

7 $$ K= \exp -{\eta}_b^2/2{H}_{\eta_b}\left({\eta}_b\right) $$

The period of an active Sun is when *2n + 1* is odd, but the tachocline is still bounded, otherwise, the series diverges because the product of an infinite series and decaying Gaussian is not bounded (that is, the Sun implodes). The results of the irradiance fluctuations during this twisting and untwisting have the feature of Hermite polynomials; namely, there is an initial global maximum and a final global minimum with the result that emissions follow alternatively active phases for *2n + 1* odd and passive phases for *2n* even (Figures 3B and C). The flipping of the polarity of the magnetic field means that there is a mirror image of the Hermite polynomial at the beginning of the next cycle (Figure 3D). Therefore, the self-similarity in emissions means that in a self-similar sequence (left to right; Figure 3D), there is a maximum in irradiance (W) at the sunspot maximum followed by the first Hermite minimum (E) and then the ‘tail’ of an error function followed by the second Hermite minimum (H), where there is higher likelihood of a sunspot minimum, before accelerating to the next W-event in the next cycle. Therefore, in this self-similar solar model there is a basic sequence of W-E-H-W in extremes of solar irradiance and, under the time fractal condition, the same sequence should occur for longer time periods in the Sun’s evolution (that is, W-E-H-W-W-E-H-W).

- In the phases transition, the solar period Ω is related to the solar radius R_S_ by the simple expression for *b* constant (which is a multiple of the fine structure constant)

8 $$ \varOmega =b\sqrt{R_S} $$

This means that significant phase transitions in the magnetic field in the model are interdependent with changes in the solar diameter.

In summary, from the physics of this matrix, there are *4n* periods of an active Sun and either *n* or *2n* periods of a passive Sun. Active phase perturbations are defined through Hermite polynomials, whilst passive phases are curvilinear error functions. Alternatively, active phases are described topologically through loops (*n*) and passive phases as either ‘loop and tails’ (*n + n*/*4*) or ‘loop and double tails’ (*n + n*/*2*). Loops with double tails (that is, where the solar minimum period is doubled) are more likely to be associated with significant magnetic events within the Hale Cycle column (*Col. j*), such as, during the Little Ice Age. At a smaller scale, there is a ‘double tail’ of the current solar minimum experienced since the solar maximum in 2002, suggesting the supposition of a centennial cycle, not seen since the previous ‘1900’ Minimum in solar activity at the beginning of the 20^th^ century.

Significant changes in solar irradiance and magnetic fields over long periods of time are also related to fluctuations in the solar diameter. Sturrock and Bertello (2010) present a power-spectrum analysis of 39,024 measurements of the solar diameter made at the Mount Wilson Observatory from 1968 to 1997 containing a number of very strong peaks agreeing closely with the frequencies of r-mode oscillations for a region of the Sun where the sidereal rotation period is 12.08 year. Qu and Xie (2013) in the study of periodicity variations of the solar radius, between 1978–2000 September found that the solar radius is in complete anti-phase with the sunspot numbers, showing an ~11 yr periodicity and a lead time of 74 months relative to the sunspot numbers. Over a longer period, such periodicities in the Sun’s solar radius could correlate with major changes in the Earth’s climate. As Rozelot (2001) notes:

“It is shown that at least over the last four centuries, warm periods on the Earth correlate well with a smaller apparent diameter of the Sun and colder ones with a bigger Sun”.

Therefore, a change in the frequency of emissions and their phase periods, defined within the harmonics in the matrix rows, are related to the rotational velocity within the core of the Sun. A smaller Sun converting more mass into energy, increases its irradiance and magnetic field activity (sunspots, solar flares), warming the Earth at the peak of loop phases, whilst an expanding Sun, occurs during the solar ‘tails’ and this is associated with a relative cooling within the Earth’s climate, for example, The Little Ice Age. The Sun, therefore, pulsates relative to the scale of the loop and tail and at the Phanerozoic scale the fluctuations are substantial. The key question becomes: do large scale positive extremes in nuclear fusion in a contracting Sun, increase its rotational rates to raise the likelihood of the potential flipping of the Sun’s dynamo orthogonally, as is the case of other stars, and in doing so, affecting the solar wind and gravitational relationship between the planets?

### An example of the model: solar emissions since the Little Ice Age

**Figure 4:**
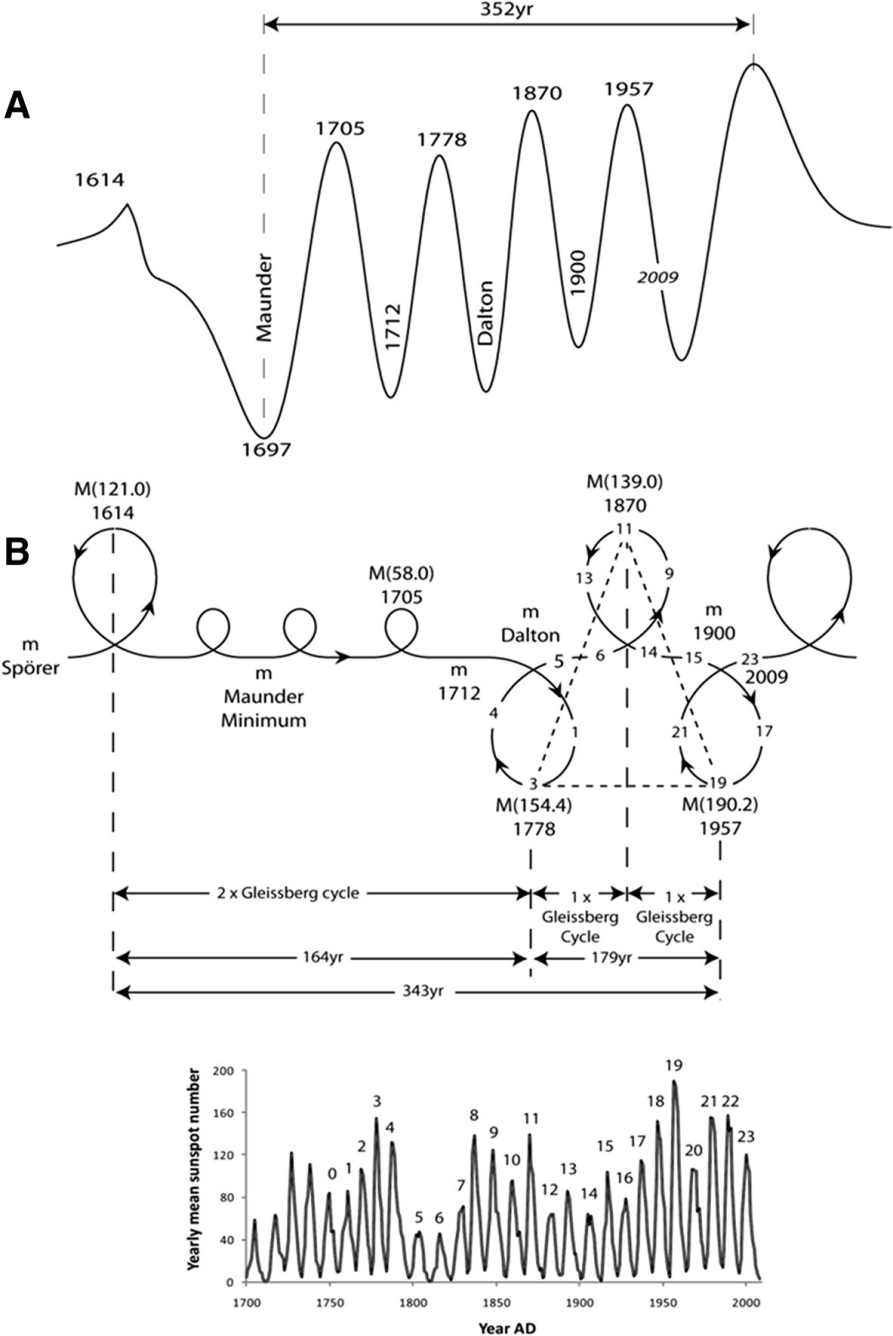
**A** The Hermite H-minimum and W-maximum irradiance sequence of the last 400 yrs, showing the H Maunder Minimum and centennial H Minimums of ‘1712’, Dalton and ‘1900 Minimum’ following the sequence of relative W peaks of sunspot maximums at 1705 AD, 1778 AD, 1870 AD and 1957 AD. **B** The loop and tail diagram of the time paths for the same sequence showing the relative sunspot maximums at the peak of the 44 yr and 88 yr loops and centennial sunspot minimums in 22 yr tails. There are four Gleissberg Cycle equivalents between the maximums at 1614 and 1957 maximums (343 yrs) which is quarter of a Bond Cycle. There is also a loop and tail symmetry between sunspot peaks 1614 AD and 1778 AD, 1778 AD to 1870 AD and 1870 to 1957 AD, all within multiples of a Gleissberg Cycle. The sunspot record for 1700 to 2009 is shown below (Source: Baker 2014).

The periodicity in sunspot formation between an active and passive Sun for the last 400 years since the Little Ice Age can be described by both methods from solutions of the equations (Figure 4A and B). There is the equivalent of two Gleissberg Cycles (~164 yr) between the Maunder Minimum period till the next sunspot maximum in 1778 AD (mean of 154.4). The Maunder Minimum is an example of a double tail in a centennial cycle. The next centennial maximum in sunspot activity is in 1870 AD (mean of 139.0) and this is again a Gleissberg period (~92 yr) from 1778 AD. This maximum in solar activity is also at the zenith of a Gleissberg loop composed of 8 sunspot cycles. The next centennial maximum is in 1957 AD (mean of 190.2) and again is one Gleissberg Cycle from 1870 AD (~87 yr), two from 1778 AD (~179 yr) and at the zenith of a Gleissberg loop. The intervening centennial solar minimums are the tails of these loops of solar activity. The reflected global minimum of solar activity in the Maunder Minimum in ~1669 AD represents the global irradiance minimum **H** in the Hermite polynomial (Figure 4A). The initial global Hermite minimum **E**, which is in phase, would be the Spörer Minimum at ~1453 AD commencing the “Little Ice Age”. This is an example of how we can sequence periodically maximums and minimums in solar activity in passive tails and then active loops to a solar maximum in 1957 AD and now the subsequent current solar centennial minimum.

The similarity matrix can also make prediction of the periodicities of the Sun not only at a decadal scale, but at centennial, millennial and million year periods. By using this self-similarity and the results of significant spectral periods found in published results (Table 1), we can construct various models of the Phanerozoic and compare the extremes in the phases with major biological, climatic and tectonic events in the geological record.

### Modelling using the self-similar matrix

The self-similarity matrix makes predictions of the periodicities of the Sun from daily to a million year time scale between higher relative frequencies in rows (R2, *1*/*α*) and (R3, *1*/*α*^*2*^) and lower relative frequencies in (R4, *1*/*α*^*3*^) and (R5, *1*/*α*^*4*^). This time fractal matrix means that the same structures implicit in the time fractal can describe the periodicity in emissions at any time scale, including the Phanerozoic. On inspection, the comparison between the spectral periods for marine diversity and extinctions (Table 1) is more closely represented by R3 predictions and they are made up principally by loop (*n*) plus double tail estimates (*n*/*2*) (Table 1). The R5 predictions are mainly loop and tail only (*n + n*/*4*) , so it is interesting to compare the predictions with the spectral periodicities for geomagnetic reversals, where R3 values predict the 12 Ma, 15 Ma, 47 Ma, 114 Ma and 285 Ma, but the R5 values appear closer to the 21 Ma, 33 Ma, and 63 Ma harmonics. So there is nothing unequivocal to suggest faster solar rotations underpin the periodicity in magnetic reversals, but there could be a mix between faster and slower loops. The question of which row is best to use in constructing the model requires further analysis. Consequently, we will analyse a sea-level data set from the mid-Cretaceous, using single taper (or filter) or multitaper (or filters) spectral analysis, to ascertain whether firstly, there are any significant periods in the data and secondly, whether sea-level fluctuations share the common periodicities found in Table 1.

Multitaper spectral analysis of the Kominz et al*.* (2008) sea-level data therefore will help in evaluation of the robustness of each row. The significant spectral densities found in the single taper Blackman (Harris) method are at 17.07 Ma (95% significance), 31.16 Ma (99% significance) and 141.55 Ma (99% significance) (Table 2). These periodicities are common with those quoted in Table 1 and it is interesting that the R5 estimates appear to be a better approximation than the R3 values (Table 2). This data is then re-analyzed using multitaper spectral analysis (MTA, BW =4 and K tapers = 5; *Autosignal*, 2007), yielding significant periods of 724,482 yr, 1.041 Ma, 2.059 Ma, 3.281 Ma and 4.887 Ma, where the four extra filters deconstruct the longer periods into equivalent sub-harmonics. The corresponding theoretical predictions for these sub-harmonics can also be calculated from Table 3 for both R3 and R5 rows. Whilst the R3 prediction of 718,336 yr is much closer to the spectral density of 724,482 yr (compared to 823,858 yr) with a higher significance (95%), there is still a spectral period of 815,729 at 90%. If we regress these theoretical values against the Miocene-Cretaceous periodicities (MCP), we will obtain an idea of which row is the better approximation, since a line of perfect correlation at 45° between theoretical values from Table 2 and the significant spectral period will have a slope of one and zero y-intercept. The results are stated for each row as:

$$ R3T=127,954+1.075MCP\kern0.5em R- squared=0.963\left(92.5\%;\kern0.5em  Theor.\kern0.5em  Error=127.95 ka\right) $$

$$ R5T = 1,806 + 0.995MCP\ R- squared = 1.00\ \left(99.995\%;\  Theor.\  Error = 1.806\  ka\right) $$

The regression diagnostics suggest that the R5 predictions overall are more strongly correlated with the MTA spectral densities. On this evidence, the best approximation of the Cretaceous to Miocene spectral data appears to be the R5 row with 99.995% prediction and a theoretical error of only 1,860 yr from a 5 Ma time period. This smaller period analysis suggests that for loop and tail, the Phanerozoic model, on balance, should be based on the R5 row values (that is, a fundamental loop of 52.75 Ma and a tail of 13.18 Ma or a 65.93 Ma period), although there is a high likelihood that there is a mixture of faster and slower rotation phases in the evolution of the Sun over this period. The conclusion via the matrix is similar to the Lieberman and Melott (2012) result using the Paleobiology Data Base (PBDB), namely, that the most significant spectral peak in this record is at 62 ± 3 Ma and we should build the model accordingly, with major sea-level fluctuations following a half loop (that is, 32.97 Ma between the cold **E-** and **H-**events and the warm **W**-events).

## Periodicities in deep time

### A solar loop and tail diagram for the Phanerozoic

**Figure 5:**
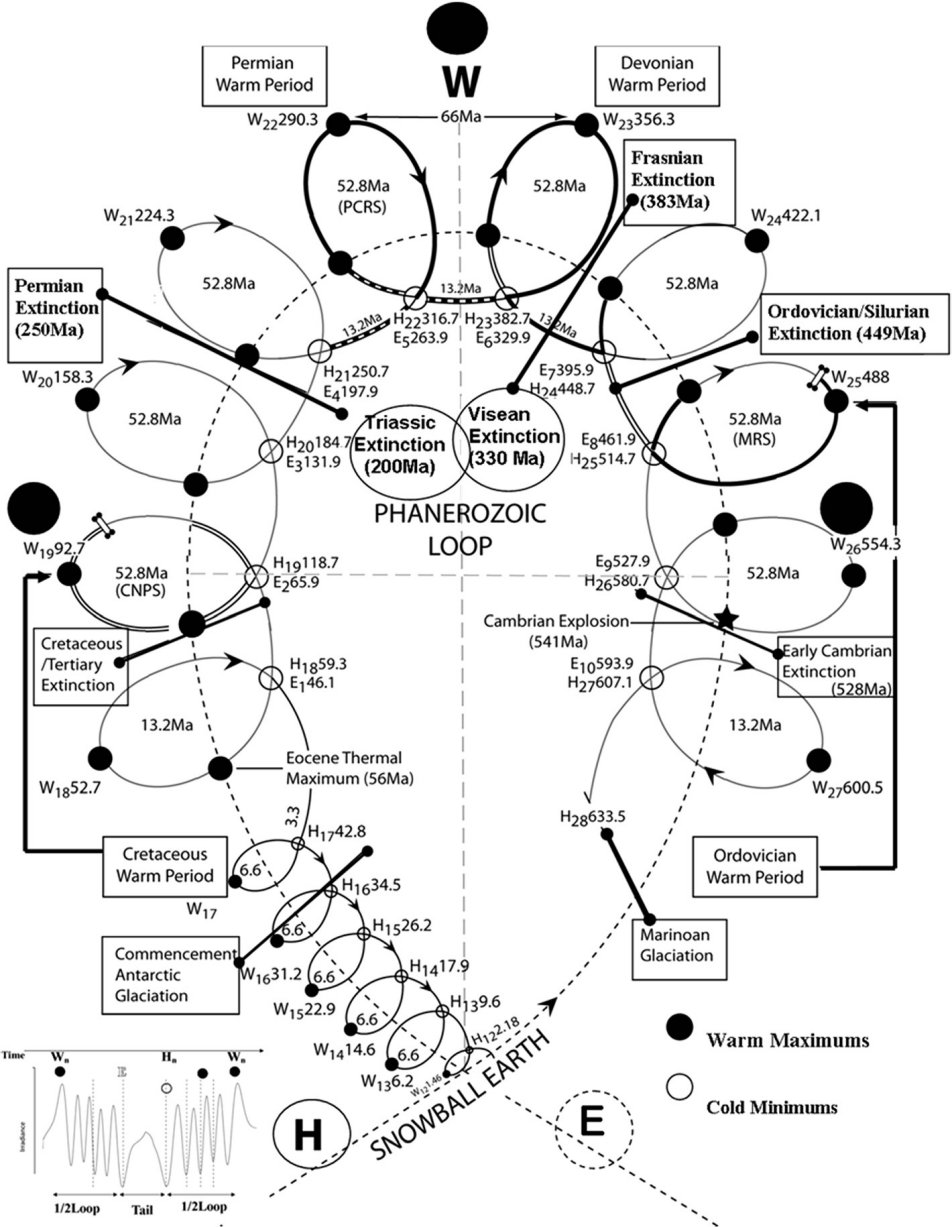
A symmetrical R5 Solar Loop and Tail Diagram for the Phanerozoic with H- and W-phases and the 66 Ma sub-harmonic sequences, showing the coincidence between E_n_-, H_n_-and W_n_-phase boundaries with major climate and extinction events. The events recorded in Table 4 can be posted to this model. The **W**-phase of the zenith of the loop coincides with Permian and Devonian Warm Periods, whereas the Ordovician and Cretaceous Warm Periods, are at the end of the first and third phases, respectively. The **H**-tail of the Phanerozoic loop defines “Snowball Earth”. Note the mirror symmetry in the mass extinction events, such as, the Permian and Ordovician at **H**_**21**_ and **H**_**24**_ and the Cretaceous and Early Cambrian at **E**_**2**_ and **E**_**9**_ and Triassic and Famennian at **W**_**21**_ and **W**_**23,**_ respectively.

This R5 periodicity is summarized in a loop and tail diagram (LTD) for the Phanerozoic where there are 52.8 Ma loops and 13.2 Ma tails (Figure 5). The **E**_**n**_ and **H**_**n**_ phases are the initial reflected global Hermite minimums that occur at the beginning and end of the passive phase of the Sun (that is, a tail), where the **H**_**n**_ phases should correspond to the coldest global temperatures (much like the Maunder minimum at the end of the Little Ice Age) and sea levels should be at their relative lowest in the cycles. The Spörer Minimum is the equivalent **E**_**n**_ Hermite minimum at the beginning of the Little Ice Age tail. At the Phanerozoic scale, the equivalent relationship occurs through fractal arguments, where the characteristic values now define four 13.2 Ma phases in a loop and one 13.2 Ma phase in the tail. The phase of maximum solar activity is at the zenith of the loop (**W**_**n**_) at the end of the second phase and this is a time of maximum solar irradiance and magnetic field twisting. In the R5 sequence, this occurs 26.4 Ma from every **H**_**n**_ phase at the **W**_**n**_ phase. These phases are the peaks of warm periods with higher sea-levels, whilst the tail boundary times, **E**_**n**_ and **H**_**n**_, define cold phases, possible glacial periods and lower sea levels.

When these solar phase extreme boundaries **(E**_**n**_, **H**_**n**_ and **W**_**n**_) are plotted on the Exxon Sea Level Curve, there appears to be good correspondence between maximum and minimum relative sea-level falls (Figure 1). The predicted **H**_**25**_, **H**_**24**_, **H**_**23**_, **H**_**22**_, **H**_**21**_, **H**_**20**,_**H**_**19**_ and **H**_**18**_ Hermite global minimums in solar irradiance at the end of the 13.2 Ma tails are times of maximum sea-level falls. These coincided with lowstands in sea levels, which were also a time of major marine extinctions (greater than 20%) at **H**_**25**_ at ~514.7 Ma in the Cambrian; **H**_**24**_ at ~448.7 Ma in the Ordovician; and **H**_**23**_ at ~382.7 Ma in the Devonian (Hallam and Cohen, 1989). This is continued with only 38% extinctions at **H**_**22**_ at ~316.7 Ma, returning to 71% extinctions at H_21_ at ~251 Ma during the Permian. The other major extinctions were at **E-**events 197.9 Ma at the Triassic/Jurassic boundary and 65.9 Ma at the Cretaceous/Tertiary boundary. These also occurred with minor negative sea-level fluctuations. These extinctions, however, preceded major sea-level falls at the **H**-events, but this relationship requires more detailed research.

The warm maximums of **W**_**25**_, **W**_**24**_ and **W**_**23**_ in the loops on the positive second phase of the Phanerozoic loop correspond to positive relative sea-level change of up ~150 m. However, the negative third phase in the Phanerozoic loop, as it descends to a point of inflection from **W**_**22**_, **W**_**21**_ and **W**_**20**_, consists of only small positive fluctuations of up ~50 m. It is only at **W**_**19**_ ~ 93 Ma ago, at the point of inflection of the Phanerozoic loop and the zenith of the Cretaceous loop, that sea levels returned to the relative positive heights of 200 m above present datum, which was not experienced since the Ordovician **W**_**25**_ warm peak at the beginning of the second phase. The sea-level curve therefore appears sensitive to the macro-dynamics of the predicted large-scale periodicities coincident with switching events in the model, which is not surprising considering the 99% significant period in the Kominz et al. (2008) sea-level data is half a loop and tail or 31.2 Ma.

The Phanerozoic loop (with an R5 period between ~600 Ma to ~660 Ma), has the same characteristics of the smaller 66 Ma loop and tails. There is an **H**-phase (greater than 635 Ma) terminating an ~165 Ma tail, which defines the end of the Cryogenian Period of the Proterozoic, 650 Ma to 850 Ma ago. The Cryogenian Period was a time when the Earth endured all-consuming Ice Ages with the evidence of glacial ice in the tropics, producing what has been termed “Snowball Earth”. This ~165 Ma tail must have been a time of a “Large Sun” and what increased the Sun’s rotation after this period is open to conjecture of how stars evolve in the Universe. What is interesting is that the end of Marinoan Glaciation at 633.5 Ma (**H**_**28**_), after the switching point of the Phanerozoic **H-**phase, is coincident with the commencement of Antarctic Glaciation at 34.5 Ma (**H**_**16**_) within the symmetry of this loop.

There are many more quai-symmetries relative to warming events, ice ages and extinction events which will be illustrated from this model. For example, the Cambrian explosion of life occurred at ~542 Ma following “Snowball Earth” and it occurred in-phase (13.2 Ma after **W**_**26**_) in the second 52.8 Ma loop of the Phanerozoic. This would have been a time of increased magnetic activity, atmospheric ionisation and temperatures. The “Early” Cambrian marine extinction event at 527 Ma is coincident with the beginning of the **E**_**9**_ phase, when there was a flipping from an active Sun to a passive Sun for the next 13.2 Ma, corresponding to a plunge in global temperatures and sea levels up to 20 m below present (Figure 1). It is also interesting that within the symmetry of the Phanerozoic loop, the “Early” Cambrian extinction event is in-phase with the Cretaceous/Tertiary extinction event at 65.9 Ma (**E**_**2**_) some 462 Ma or 7 Cycles later. Likewise, the major Ordovician/Silurian extinction at 448 Ma (**H**_**24**_) is symmetrical around a 90° axis with the major Permian Mass Extinction event at 250 Ma (**H**_**21**_).

The Devonian Warm Period maximum at 356.3 Ma (**W**_**23**_) and the Permian Warm Period maximum at 290.3 Ma (**W**_**22**_) continue 66 Ma phase periodicity. They are at the **W**-peak of the Phanerozoic loop and in phase with the end of the “Snowball Earth” tail at **H**. The Ordovician Warm Period at 488.7 Ma (**W**_**25**_) and Cretaceous Warm period 92.7 Ma (**W**_**19**_) are at the zenith of 52 Ma loops and are also close to points of inflection in the larger Phanerozoic loop. The onset of the Ordovician Glacial period in the 461.9 Ma to 448.7 Ma tail (**E**_**8**_ to **H**_**24**_), Carboniferous Glacial Period 329.9 Ma to 316.7 Ma tail (**E**_**6**_ to **H**_**22**_) and the Early Cretaceous Cold Period 131.9 Ma to 118.7 Ma tail (**E**_**3**_ to **H**_**19**_) are all internally consistent within the Phanerozoic loop.

The beginning of the Cretaceous to present (Phase 4) is symmetrical to Phase 1 loops and tails, which preceded the Cambrian Explosion (~540 Ma) and extended to the Cryogenian (the end of “Snowball Earth”). This makes direct comparison for the future of Earth problematic from the paucity of information, although the commencement of Antarctic glaciation at ~34 Ma (H16) and the end of Marinoan glaciation at ~634 Ma (H28) have some reflected symmetry.

The Permian Mass Extinction (**H**_**21**_) and the Triassic Mass Extinction (**E**_**4**_) also form a 52.8 Ma loop of extinction. This axis of extinction has some symmetry with the Frasnian (**H**_**23**_ -382.7 Ma) and the Visean (**E**_**6**_- 329.9 Ma) axis in the Devonian-Carboniferous loop on the right-hand lobe, but is not as severe as the former loop. Indeed, the degree in **H** and **E** extinction severity appears to change between the left-hand and right-hand lobe of the Phanerozoic loop.

The Mass Extinction events of the right-hand lobe of the Phanerozoic are **H**-events (Cambrian **H**_**25**_**,** the Ordovician **H**_**24**_ and the Frasnian **H**_**23**_ ), whereas after the Permian (**H**_**21**_ ), the next two on the left-hand lobe are **E**-events (Triassic **E**_**4**_) and Cretaceous (**E**_**2**_). The corresponding **H**-events at **H**_**20**_, **H**_**19**_ and **H**_**18**_ are much less severe with less than 26% genera disappearing compared to Phase 2 and 3 where the range for **H-**events were between 38%-74% with an average of 59.6% (Sepkoski, 2002). Likewise, there has been no major extinction event with the present **H**-event (that is Pleistocene Ice Ages) and little biological information can be discerned from Phase 1, since events preceded the Cambrian explosion.

The frequency of geomagnetic reversals has also changed between lobes. Idnurm et al. (1996) compared the 70 Ma Ordovician reversal pattern (right loop lobe) with the Cainozoic reversal pattern (left loop lobe), showing the Ordovician frequency is ~5 Ma compared to the Cainozoic frequency of ~0.5 Ma. It is interesting that phases of this magnitude (that is ~5 Ma) are in the right-hand lobe where normal to reverse polarity flips occurred during the Ashgill Epoch (443-434 Ma) at **H**_**24**_ coinciding with Ordovician Mass Extinction (61%), whilst the Frasnian Epoch (384-372 Ma) at **H**_**23**_ (382.7 Ma) preceded the flipping to reverse polarity in the Frasnian (378-369 Ma) that opened the doorway to the Frasnian Extinction (54%). The Frasnian extinction ended at the possible intersection of the Phanerozoic loop with the Devonian-Carboniferous 52.8 Ma loop and may explain why there is only 13.2 Ma apart from the significant **W**_**23**_ Famennian extinction (Figure 5). The global Hermite minimum at **H**_**22**_ at 316.7 Ma is also consistent with this sequence, since it was at the beginning of the switch from normal to reverse polarity in the Permo-Carboniferous Reverse Superchron and was also a time of significant extinction (38%) during the Narmurian Epoch (325-314 Ma). The flip from the normal polarity from the Dorashamian Stage (255-251 Ma) was followed by the Permian Mass Extinction (71%) at **H**_**21**_.

For **W-**events, there is a flipping period from normal to mixed polarity at **W**_**24**_ (422.1 Ma) during the Wenlock Epoch in the Silurian and this was followed by the Ludlow Extinction (40%). At **W**_**23**_ (356.3 Ma), there was a change by 354 Ma from normal polarity to reverse polarity for the entire Tournaisian, concurrent with the Famennian extinction (49%). The **W**_**25**_ event (488 Ma) at the time of the Cambrian-Ordovician extinction (57%) occurred with a flipping of normal polarity to reverse polarity for the entire Arenig Epoch (486 Ma-465 Ma).

The last major geomagnetic reversal (Brunhes–Matuyama reversal at 780,000 yr) lies between the R3 718,336 and R5 823,858 yr spectral density periods (Table 2) and 769,704 yr R4 value (Table 3). The previous major reversals, namely: Matuyama (2.59 Ma), Gauss (3.59 Ma) and Gilbert (5.25 Ma) closely approximate an R3 loop and tail sequence (using an R3 sequence constructed with 89,702 yr and 179,404 yr loops; and 134,553 yr, 269,108 yr and 538,212 yr loop and double tails). A geomagnetic reversal from normal to reverse polarity, associated with the H-event, is highly likely; however, there is still uncertainty as to what loop period defines the mid to late-Pleistocene. If it is an R3 loop, then the reversal was likely after 50,000 yr BP, for R4 at 10,000 yr BP and for R5, flipping in the future at 26,000 yr. The event at this scale should first have an **E**-, **H**-or **W**-event and this could have already occurred between 14,500 and 11,500 yr BP with the significant Bølling-Allerød interstadial-Younger Dryas glacial ~15°C oscillation of sudden warming to present and then a return to the Ice Age. As a **W**-event, the Holocene would represent a ~10,000 yr interstadial period before an expected **E**-event. Over a longer period, there has been no geomagnetic reversal (normal to reverse) in an **E-** or **H**-event in an ~760,000 yr Pleistocene loop sequence. Likewise, there has been no mass extinction event yet recorded terminating the Pleistocene **H**-period of the last 66 Ma Phanerozoic loop. The situation in the geological ‘now’ presents a convergence of possibilities.

### Biological, climate, geomagnetic and tectonic events in the Phanerozoic

Much of the biological, climatic, geomagnetic and tectonic events (Young and Laurie, 1996; Haq and Schutter, 2008; Sepkoski’s Compendium, 2002) can be posted to the loop and tail diagram and the clustering of these events around the major **W-**, **E-** and **H-**phase change boundaries is quite striking (Table 4). These events will be summarised in the following sections compared to these boundaries.

**Table 4:** **Comparison with the R5 Loop and Tail Solar Model with Geomagnetic, Biological and Geological Events (Sources: Young and Laurie,** 1996 **; Haq and Schutter,** 2008 **; Sepkoski’s Compendium,** 2002 **)**

***Phase***	***Phase time Ma***	***Event limit***	***Geological time***	***Biological and Geological Event***
		**31.0**	**Rupelian C12**	
**H**_**16**_	**34.5**			**• Commencement of Antarctic Glaciation**
				**• Mass Extinction (34-23Ma: 21% Genera)**
**E**_**1**_	**46.1**	**46.6**	**Lutetian C21**	**• Paleocene-Eocene Thermal Max**
_**(Phase 1 13.2Ma Loop)**_	**56.0**	**55.0**		**• Mass Extinction (55.8-33.9:Ma 31% Genera)**
**E**_**2**_	**65.9**	**65.5**	**29R Maastrichtian**	**• N-R Polarity Reversal**
				**• Major Cretaceous/ Tertiary Extinction (65.5Ma: 47% Genera)**
**W**_**19**_	**92.7**	**83**	**Cenomanian-Turonia**	**• Extinction (94-89Ma: 27% Genera)**
				**• Cretaceous Warming Maximum**
**H**_**19**_	**118.7**	**119**	**Aptian** ***Cretaceous Normal Polarity Superchron***	**• Extinction (125-112Ma: 19% Genera)**
			**M3r Barremian**	**• R-N Polarity Reversal (119Ma-83Ma)**
**E**_**3**_	**131.9**	**131**	**M11r Valanginian**	**Late Jurassic Warm Period**
**W**_**20**_	**158.3**		**Meneghinii**	**• Late Jurassic Extinction (161-150Ma: 19%-32% Genera)**
**H**_**20**_	**184.7**	**184.6**	**Toarcian**	**Extinction (183-177Ma: 24% Genera)**
**E**_**4**_	**197.9**	**195**	**Jamesoni**	**• Major Triassic Extinction (200Ma: 63% Genera)**
				**Triassic /Jurassic Cold Period**
				**• N-R Polarity Flip**
**W**_**21**_	**224.3**		**Norian**	**• Norian Extinction (228Ma: Genera 39%)**
				**• Triassic Warm Maximum**
**H**_**21**_	**250.7**	**252**	**Dorashamiam/Changsing**	**• Second Major Permian Extinction (260-251Ma: 71% Genera)**
				**• N-R Polarity Reversal**
				**• Major Orogenic Events**
				**Pflaazian, Palatinian, Sonoma 251** ***±*** **5Ma**
				**• Phanerozoic sea-level minimum (−40m MSL)**
				**Anoxic interval 260–250 Ma**
**E**_**5**_	**263.9**	**262**	**Midian**	**• First Major Permian Extinction (271-260Ma: 56% Genera)**
			***End of Permo-Carboniferous Reverse Superchron***	**• Reverse Superchron (305Ma-263Ma)**
				**• Phanerozoic sea-level minimum (−60m MSL)**
**W**_**22**_	**290.3**			**• Extinction (280-271Ma: 33% Genera)**
				**• Phanerozoic sea-level change (+100m) Permian Warm Period**
**H**_**22**_	**316.7**		**Narmurian**	**• Extinction (325-314Ma: 38% Genera)**
				**• Early Whichita Orogeny 318** ***±*** **5Ma**
				**• Late Whichita Orogeny 310** ***±*** **5Ma**
				**• Phanerozoic sea-level minimum (−70m MSL)**
**E**_**6**_	**329.9**	**325**	**Visean**	**• Glaciation Commences ~330Ma**
				**• Major Orogenic Events: Sudetian Orogeny 325** ***±*** **5Ma**
				**• Extinction (45% Genera)**
**W**_**23**_	**356.3**	**354**	**Famennian**	**• Famennian Extinction (375-359Ma: 49% Genera)**
				**• Devonian Warm Maximum (356 Ma)**
				**Svalbardian Orogeny 360Ma** ***±*** **5**
				**Antler Orogeny 364Ma** ***±*** **5**
**H**_**23**_	**382.7**	**378**	** Frasnian**	**• Frasian Mass Extinction (383-374Ma: 54% Genera)**
				**• Polar Reversal N-R (378Ma- 356Ma)**
				**• Glaciation ~380Ma**
				**• Anoxic interval (380 & 360 Ma)**
**E**_**7**_	**395.9**	**390**	**Eifelian**	**• Extinction (398-392Ma: 38% Genera)**
				**• Major Orogenic Events:**
				**Ebrian 400Ma**
				**Hibernian 403Ma**
**W**_**24**_	**422.1**		**Ludlow**	**• Extinction (423-419Ma: 40% Genera)**
				**• Phanerozoic sea-level maximum (+200m)**
**H**_**24**_	**448.7**	**451 (N/R)**	**Ashgill**	**• Second Major Ordovican Extinction (449–444 Ma: 61% Genera)**
				**• Magnetic (N-R) reversal 451Ma**
				**Taconian Orogeny 449** ***±*** **5Ma**
				**• Anoxic Interval-443Ma**
**E**_**8**_	**461.9**		**Caradoc** ***End Mayero Reverse Superchron***	**• First Major Ordovician Extinction (458-449Ma: 44% Genera)**
			**Arenig**	**• End Superchron (486Ma-465Ma)**
				**• Sardininian Orogeny (461.9** ***±*** **5Ma)**
				**• Phanerozoic sea-level minimum(460Ma)**
**W**_**25**_	**488.0**	**486**	***Begin Mayero Reverse Superchron***	**• Cambrian/Ordovician Extinction (490-485Ma: 57% Genera)**
			**Tremandoc**	**• Delhi Orogeny (488** ***±*** **5Ma)**
				**• Begin Superchron (486Ma-465Ma)**
				**• Ordovician Warm Maximum (488Ma)**
				**• Anoxic Interval (26Ma of Anoxic Conditions (514.7Ma to 488Ma)**
				**• Phanerozoic sea-level maximum (+240m)**
**H**_**25**_	**514.7**	**511**	**Precedes Upper Toyonian limit**	**• Second Cambrian Extinction (520-513Ma:74% Genera)**
				**• Pampean Orogeny (514.7Ma** ***±*** **5)**
				**• PolarityFlip (R-N)**
**E**_**9**_	**527.9**	**528**	**Lower Toyanian Boundary**	**• First Cambrian Extinction (530-520Ma: 62% Genera)**

### Paleozoic Era (545-246 Ma ago)

At the beginning of this Era, the continents were far apart but plate tectonic processes moved together into one large supercontinent called Pangaea after a significant **H**-event and field flipping. The end of the first Cambrian loop was the Hermite minimum **E**_**9**_ (527.7 Ma) that coincided with the R-N polarity flip at the lower Toyanian boundary at 528 Ma. This is within the range of the commencement of the Cambrian extinction event at 530 Ma. Extinctions peaked towards the end of the tail where, by 520 Ma, 74% of taxa had disappeared. This was also a time (521-511 Ma) of high frequency sea-level fluctuations, when sea levels fell a number of times to ~60 m below present datum. The **H**_**25**_ Hermite minimum at 514.7 Ma at the end of the tail was accompanied by a flip in polarity (R-N) at ~511 Ma and the Pampean major orogeny.

The first lobe of the next loop beginning at **H**_**25**_ and terminating at the zenith **W**_**25**_ at 488 Ma was a period of 26 Ma of anoxic conditions in the oceans. Sea levels rose to ~200 m above present, although there was a significant oscillation at 495 Ma. This was also the time of the Delhi major orogenic event. The next 26 Ma was period of a possible reverse geomagnetic Superchron (486 Ma to 465 Ma) and covers the entire period of the second lobe of the Ordovician loop **W**_**25**_ to **E**_**8**_ (461 Ma). This reverse polarity was repeated (~420?Ma to 390 Ma) and is in the same negative phase of the next Silurian/Devonian loop (**W**_**24**_ to **E**_**7**_ or 422.1 Ma to 395.9 Ma). As with the Cambrian, the positive lobe of the loop, defined by the Silurian, had an extended period of anoxic conditions in the oceans between 440 Ma to 428 Ma terminating at **W**_**24**_, which was also a time of 40% mass extinctions and a major N-R reversal.

Previously, at the end of the Ordovician tail (**H**_**24**_ at 448.7 Ma) was the major Ordovician extinction event, where 987 (61%) taxa disappeared. This was the time of the major Taconian orogeny, extensive glaciation and where sea levels dropped some 75 m. This catastrophic phase was introduced by an N-R magnetic flipping at ~451 Ma during the Caradoc Epoch.

The positive lobe of the Devonian/Carboniferous loop (**H**_**23**_ to **W**_**23**_) was in a phase dominated by reverse polarity (378 Ma to 356 Ma). After the predicted **H**_**23**_ irradiance minimum at 382 Ma, there was the commencement of a series of short glacial periods till 350 Ma where sea levels were relatively stable, until the significant perturbation at 359.2 Ma at the Famennian boundary at the end of the Devonian, where sea levels rose to be near present (Haq and Schutter, 2008). This coincides with the **W**_**23**_ maximum in irradiance and the Devonian Warm Period maximum (356.3 Ma). Like previous loops, there were anoxic conditions in the oceans after **H**_**23**_ and before **W**_**23**_. The **H**_**23**_ irradiance minimum was also a time of the Frasnian Mass Extinction (385.3 Ma) where 54% of taxa became extinct and its conclusion was coincident with an N-R reversal at ~378 Ma. The R-N reversal at ~356 Ma was also a time of major orogenic events (namely, the Bretonian and Svalbardian) and this reverse polarity mostly continued for approximately the next 28 Ma until its termination at the end of the Visean Epoch at **E**_**6**_**.** This coincided with the Sudetian major orogeny.

The Permo-Carboniferous Reverse Superchron (305 Ma to 263 Ma) covers most of the loop (**H**_**22**_ to **E**_**5**_ or 316 Ma to 263 Ma) at the peak of the Phanerozoic loop. It was a time of extensive glaciation, but also interrupted by a warm spike (**W**_**22**_ at 290.3 Ma) when sea levels were ~59 m above present. The glacial maximum period was, however, between **E**_**5**_ at 263.9 Ma and **H**_**21**_ at 250.7 Ma, when sea levels were ~100 m below present, the lowest of the Paleozoic. This tail was a period of normal polarity that flipped back to reverse fields 252 Ma ago. It was also an occasion for a major mass extinction event, where 71% of taxa suddenly became extinct by 250 Ma. By this time, there were only 255 taxa remaining on Earth, a loss of 983 taxa, where 80% had vanished since 270 Ma. The **H**_**21**_ period at 250 Ma was also a time of major orogenic events (Pflaazian, Palatinian, Sonoma and Hunter-Bowen). The major Permian and Ordovician extinction events are symmetrical to the axis of the Phanerozoic loop and in-phase 198 Ma apart (or 3×66Ma loop and tails) (Figure 5).

### The Mesozoic Era (246-65 Ma ago)

At the commencement of the Mesozoic, there was the continental landmass Pangaea. However, during this Era, the continents pulled part and commenced to drift across the Globe. The first loop of the Mesozoic ended at **E**_**4**_ period at 197.9 Ma, where there was a Hermite minimum in irradiance and sea levels fell at **H**_**20**_ at 184.7 Ma to the low stands of Carboniferous levels. Concurrently at **E**_**4**_ (197.9 Ma) there was a switch to reverse polarity at ~195 Ma and, as previously, there was a major Triassic Mass Extinction event, where by 200 Ma, 63% of taxa had disappeared. By 196.5 Ma there were only 496 remaining, a loss of 508 taxa.

The Cretaceous was a time when temperatures were 5° to 6° warmer than present, anoxic conditions persisted for extended periods and sea levels rose to ~200 m above present datum, levels not seen since the Cambrian. In the positive lobe of the loop (**H**_**19**_ to**W**_**19**_), the Cretaceous Normal Polarity Superchron is found (119 Ma -83 Ma). The famous dinosaur extinction event occurred 65.5 Ma, coinciding with the Hermite boundary at **E**_**2**_ (65.9 Ma) and whilst controversial, it was a time of polar reversal (29R N-R during the Maastrichtian) and is symmetric with the **E**_**9**_ event of the Cambrian, some 462 Ma previously (or 7×66Ma loop and tails).

### The Cenozoic Era (65 Ma to present)

The end of the Cretaceous at **E**_**2**_, was the beginning of a 13.2 Ma tail ending at **H**_**18**_ at 59.3 Ma. There was a likely change in phase to smaller 13.2 Ma loops and 3.3 Ma tails as the Phanerozoic loop descended past the point of inflection. At the first phase of this loop at 56.0 Ma is the Paleocene-Eocene Thermal Maximum where for 170,000 yr, sea surface temperatures were 8° to 10° warmer (Zachos et al. 2003) and sea levels rose to above 125 m above present. There were two relevant magnetic reversals N-R at 56.1 Ma and R-N at 55.4 Ma but nothing to suggest the reason for the extreme event, unless it was a major switching event within the Phanerozoic loop. This phase of global warming had a massive injection of isotopically light carbon into the ocean atmospheric system (Röhl et al. 2007). There was also a significant mass extinction event where 1370 taxa (31%) disappeared, but this was at the end of the first phase rather than a **W-**event.

However, the commencement of major Antarctic glaciation occurred at **H**_**16**_ (34.5 Ma) at the Eocene-Oligocene boundary and was in-phase with the past. During this period of a global minimum in irradiance, sea level fell ~ 55 m and 1227 taxa (21%) became extinct, once again associated with the beginning of an N-R chron after 33 Ma (C13) lasting for 2 Ma (Miller et al. 2008).

The last 2.18 Ma (The Quaternary Period) saw the commencement of the Quaternary Ice Ages in an **H**-event and this was a time when ice sheets periodically waxed and waned in response to the Earth’s orbital factors at frequencies of 23,000 yr, 41,000 yr and 100,000 yr. However, the periodicities from the Multitaper analysis of Cretaceous/Tertiary sea levels were still in phase with the predicted longer solar cycles (for example, 724,482 yr) and the same spectral analysis from deep sea cores yielded significant 736,000 yr as well as 95,115 yr, 41,000 yr and 23,413 yr (Thomson 1990). Therefore, a pulsating Sun and internal rotations of its core dynamo through a cycle of situations could affect the precision, obliquity and eccentricity of the Earth and its orbit (Milankovitch Cycles).

### The Hermite model of the Phanerozoic

**Figure 6:**
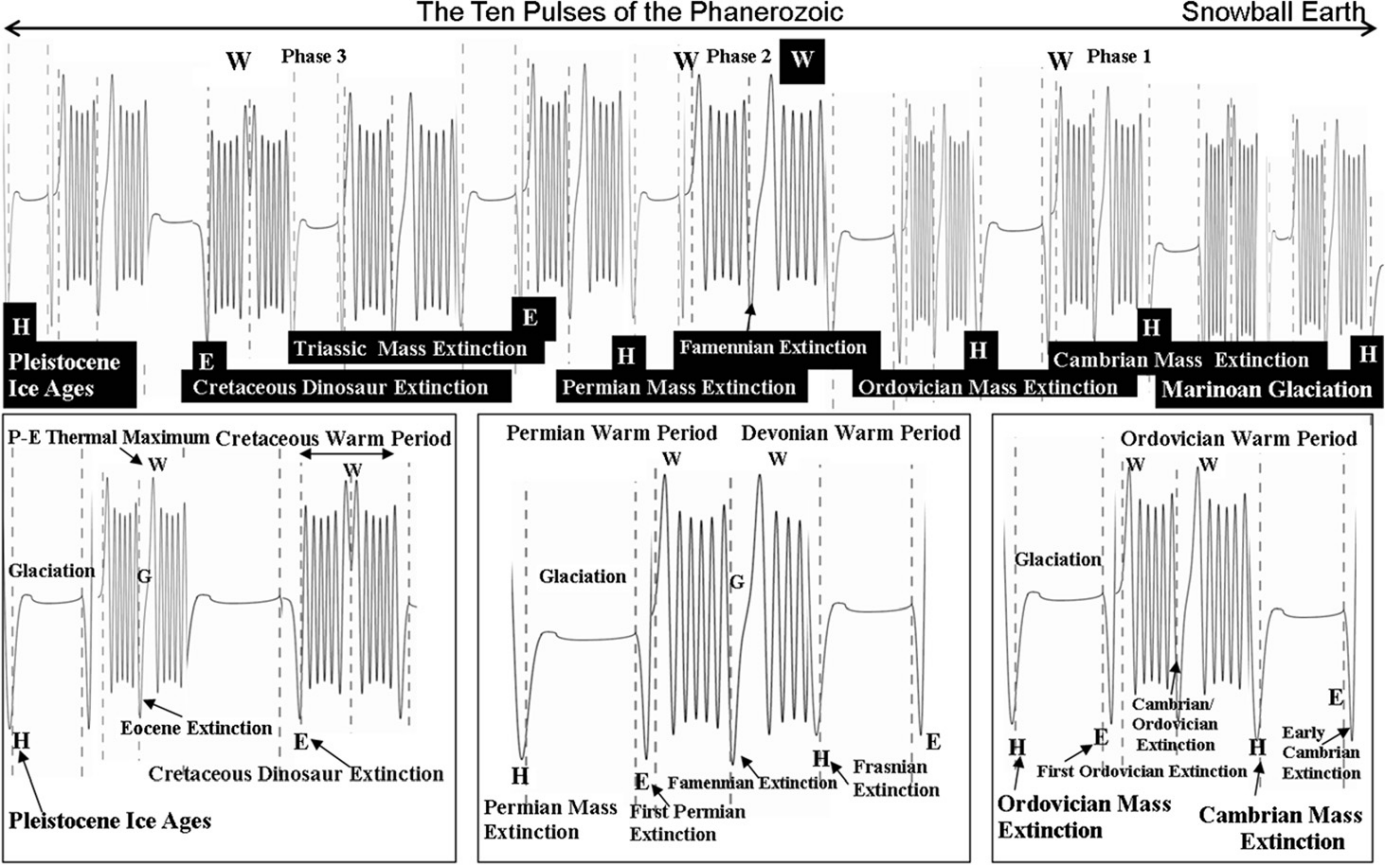
A representative Hermite model equivalent of the loop and tail diagram in Figure 5 where there are at least ten major pulses from the Sun affecting the Earth: the major extinction events are at the major phase changes, the maximum warming periods coincide with W-events and the glaciations are in tails defined by E-events and H-events. The major extinction events coincide with the flipping between the loops and tails and visa versa (that is, **H**-events and **E**-events) and at the solar maximum **W**-events. The insets show the details of parts of the sequence including the glaciation periods and phases (G) and their relationship to extinction events.

The solutions of the self-similarity DE can also be presented firstly, by Hermite polynomials and their mirror images around phase boundaries for an active Sun and secondly, by error functions for a passive Sun, similar to the model of irradiance since the Little Ice Age (Figure 4). The global maximums and minimums in the polynomials determine the timing and symmetry of extremes in warming events, glaciations, sea-level fluctuations and extinctions in Table 4. The model summarises the dynamics of the Phanerozoic, where the period is defined by at least ten self-similar pulses from the Sun. This self-similarity is highlighted by the latter two insets (Figure 6) from the Cambrian-Ordovician and Devonian-Permian periods for Phase 2 and Phase 3, respectively, where there is a common order in events of varying magnitude, namely:

**H** extinction- glaciation- **E** extinction - warm period - **W** extinction - **H** extinction (left/right)

The magnitude of these events is very much determined by where the 66 Ma loop and tail lie in the context of the points of inflection of the Phanerozoic loop (and tail), and whether the event occurs on the right-hand or left-hand lobe of the loop.

The Hermite version also allows for the extinction events to be classified according to what phase boundaries they are associated, namely:

- ***H-events****: Permian Mass Extinction (71%); Ordovician Mass Extinction (61%); Cambrian Mass Extinction (74%); Frasnian Extinction (54%); Narurian Extinction (38%)*
- ***E-events****: Dinosaur Mass Extinction (47%); Triassic Mass Extinction (63%); First Permian Extinction (56%); First Ordovician Extinction (44%); Early Cambrian Extinction (57%); Visean Extinction (45%); Eifelian Extinction (38%)*
- ***W-events:****Famennian Extinction (49%); Cambrian*/*Ordovician Extinction (57%); Ludlow Extinction (40%); Eocene Extinction (32%)*

The question of the Earth’s geomagnetic reversals at this scale can only be discussed generally with some phases having, at best, a prevalence of a particular polarity. Nevertheless, some interesting observations can be made at this scale related to the model (Table 4). The Cretaceous Normal Polarity Superchron (119 Ma-83 Ma) occurs broadly at the point of inflection between the third and fourth phase of the Phanerozoic from **H**_**19**_ to **W**_**19**_ for a period of 36.0 Ma. Subsequently, its reverses after **W**_**19**_ for 4.2 Ma (R5 sub-harmonic of loop of 3.296 Ma + 0.824 Ma = 4.12 Ma; Table 3) before flipping back to normal polarity for much of 17.5 Ma before the dinosaur extinction at **E**_**2**_, to complete one R5 loop of ~53.0 Ma. Conversely, Permo-Carbonifereous Reverse Superchron (312 Ma-262 Ma) at the zenith of the Phanerozoic loop (between the second and third phase) broadly commences at **H**_**22**_ with reverse polarity with one possible 1 Ma normal reversal at 298 Ma, preceding **W**_**22**,_ before switching to reverse polarity to the **E**_**5**_-event at 262 Ma. The R5 sequence for these phases are 14 Ma +1 Ma + 36 Ma = one R5 loop of 51 Ma. Both Superchrons are at major Phanerozoic phase changes and are broadly the polarity mirror image of each other in terms of an H-W-E sequence of events. The comparison can be broadly summarized as:

#### Cretaceous Normal Superchron



#### Permo-Carbonifereous Reverse Superchron



At the point of inflection between Phase 1 and 2 of the Phanerozoic loop, there is some evidence of a Reverse Superchron in the Ordovician between 486 Ma (**W**_**25**_) and 465 Ma (**E**_**8**_), a period 6.0 Ma of short reversals (465 to 458 Ma) and a period of normal polarity from 458 Ma to 450 Ma (**H**_**24**_). This is followed by a phase of reverse polarity for the lower Caradoc between 450 Ma and 443 Ma (or equivalent to half a 13.2 Ma tail) flipping to normal polarity till the end of the Ordovician at 435 Ma (Ashgill Epoch). The incidence of the Superchrons in the geological record seems to broadly occur in each phase of the Phanerozoic loop, but does not necessarily have any direct impact on the severity of extinction events. It looks at this stage to be a function of geomagnetic reversals in the 66 Ma and below sub-harmonics in combination with other factors.

## Conclusion

The symmetry and periodicity of this R5 loop and tail diagram and the physical Hermite model, both predict many of the climatic, geological and biological events recorded in the geological record. The juxtaposition of major events is striking, supporting the consistency in the spectral analysis of the plethora of proxies. Whether the Sun, as a maturing star, can still flip its core magnetic fields latitudinally and meridianally like younger stars, is theoretically possible but still speculative. However, the synchronous switching of this dynamo with the Earth’s geomagnetic field, in phase with fluctuations in the solar diameter and changing gravitational influences in the solar system, could provide the opportunity, during lag periods, for the Earth to be bombarded periodically with extremes in irradiation, UV-B radiation, galactic cosmic rays and an increase in the likelihood of the number and penetration of meteorites to the Earth’s surface. This amalgam will also lead to concurrent internal mantle and climate responses, which in combination with the external galactic contribution, could significantly affect, during these phase switching events, the viability of terrestrial and marine life on Earth. This periodicity is therefore underpinned by a common mechanism and extinctions not the result of random perturbations. It is postulated here to have an origin from the harmonic gyrations of the dynamo within the core of the Sun maintaining a fixed energy output as it evolves as a star. The Bølling-Allerød-Younger Dryas oscillation is not the last or the unprecedented event of the recent past, but rather part of an unfinished sequence from time immemorial: this is the quintessential lesson from Deep Time to the current Anthropogenic world.
